# Impact of Nanopore Flow Cell Types on DNA Methylation Detection

**DOI:** 10.17161/sjm.v2i2.23664

**Published:** 2025-02-03

**Authors:** Xianglin Shi, Xiaodong Lu, Xinyue Chen, Shaojun Yu, Rebecca S Arnold, Viraj A. Master, Jonathan C. Zhao

**Affiliations:** 1 Department of Urology, Emory University School of Medicine, Atlanta, GA 30322, USA;; 2 Department of Human Genetics, Emory University School of Medicine, Atlanta, GA 30322, USA;; 3 Winship Cancer Institute, Emory University School of Medicine, Atlanta, GA 30322, USA;

**Keywords:** Nanopore, DNA methylation, third-generation, sequencing

## Abstract

Third-generation sequencing technologies have revolutionized the study of epigenetic characteristics in human diseases, with Oxford Nanopore Technologies (ONT) at the forefront of long-read sequencing. ONT has made rapid improvements in flow cell designs, which greatly increased its sequencing accuracy but, at the same time, led to some projects utilizing different flow cell types, mainly R9 vs. R10, across samples. Whether and how the flow cell types affect genome-wide DNA methylation detection remains incompletely understood. Here, we used both flow cell types to analyze 6 human renal cell carcinoma (RCC) samples and compared the results. While there was a highly significant correlation between 5-methylcytosine (5mC) detected by R9 and R10 flow cells, we also observed substantial differences. R9 flow cells over-estimated 5mC levels at hypomethylated chromatin regions, mostly at promoters, while under-estimated 5mC at hypermethylated chromatin regions, enriched at intronic and intergenic regions. Such deviations in detection were likely caused by substantially lower sequencing accuracy of R9 flow cells, due to its mechanics, especially having problems sequencing homopolymeric DNA elements, such as CpG islands, leading to both higher false-positive and false-negative detections. Interestingly, such systematic errors were largely mitigated by batch-correction software, improving data comparability. In summary, our study reports superior performance of R10 flow cells, leading to much higher accuracy in base sequencing and DNA methylation detection.

## Introduction

DNA methylation, such as 5-methylcytosine (5mC) involving the addition of a methyl group to the fifth carbon of cytosine, is one of the most extensively studied DNA modifications in mammals. It plays a crucial role in regulating gene expression and genome stability in normal cells. Aberrant DNA methylation, by contrast, is a hallmark of cancer, contributing to tumor initiation and progression. Studies have shown that promoter hypermethylation represses the expression of tumor-suppressor genes, thereby increasing cell proliferation, while global hypomethylation makes the genome unstable, leading to DNA damage and promoting malignant transformation[1, 2].

Traditionally, DNA methylation has been analyzed using bisulfite-based approaches, such as bisulfite sequencing (BS-seq), a technique that first converts unmethylated cytosines to uracil to distinguish them from methylated cytosines, followed by subsequent PCR, microarray hybridization, or next-generation sequencing [3]. However, bisulfite treatment causes DNA damage, fragmentation, and loss, resulting in biased data. To address these pitfalls, enzymatic Methyl-seq (EM-seq) was developed [4]. It first uses TET2 enzymatic activity to oxidize methylated cytosines, including 5mC and hydroxymethylated cytosines (5hmC), followed by APOBEC2 to convert unmethylated cytosines to uracils. In the subsequent PCR amplification, the oxidized and methylated cytosines, i.e., 5mC and 5hmC, will generate normal base pairs with guanines, whereas the uracils, derived from unmethylated cytosines, will form base pairs with adenines, resulting in similar DNA sequence profiles as that in BS-seq [4]. EM-seq significantly increases sequencing accuracy compared to BS-seq, however, its ability to detect methylation patterns over long genomic intervals, discern haplotype-specific methylation, and analyze 5mC and 5hmC on the same reads simultaneously remains largely constrained.

The advent of third-generation sequencing technologies, such as Oxford Nanopore long-read sequencing (ON-LRS), has revolutionized DNA methylation analysis. ON-LRS enables the sequencing of native DNA strands as long as over 1 Mb with 10kb as average [5]. Additionally, it can directly detect multiple DNA modifications, including 5mC and 5hmC, concurrently without additional chemical or enzymatic conversion, and significantly reduces the time for sample library preparation [6]. Meanwhile, this technology also expands the scope of epigenetic studies by enabling haplotype-specific phasing and allele-specific methylation analysis. The ON-LRS is being rapidly developed with quick implementation and improved chemicals and flow cells. Recent advancements in ON-LRS include improvements in flow cell design. The R9 flow cell has been widely used but it has low sequencing accuracy at homopolymeric DNA regions [7]. The new version of the flow cell, R10, addresses these limitations using a dual-reader head design with an extended barrel, thereby significantly increasing sequencing accuracy at homopolymeric regions, and improving overall sequencing fidelity. As many labs have been utilizing the R9 flow cell and now starting to use the R10 flow cell as the R9 flow cell will be discontinued in the future, it is necessary to evaluate performance and data reproducibility across the two types of flow cell. A recent study took some initial steps to address these issues, however, this study only focused on one cell line and did not investigate 5mC profiles measured by both types of flow cells systemically [8].

In this study, we performed NO-LRS in 6 human kidney cancer samples using R9 and R10 flow cells and systemically evaluated 5mC profiles detected by both flow cells. We found substantial inconsistency in DNA methylation profiles measured by two versions of flow cells, which can be corrected by computational approaches. The difference is primarily caused by background noise and ambiguous signals of the R9 flow cell, leading to respective over-detection and under-detection of 5mC level at hypomethylated- and hypermethylated-genomic regions, in comparison to the R10 flow cell.

## Results

### Systematic differences of R9 and R10 flow cells in detecting 5mC

**Figure 1.**
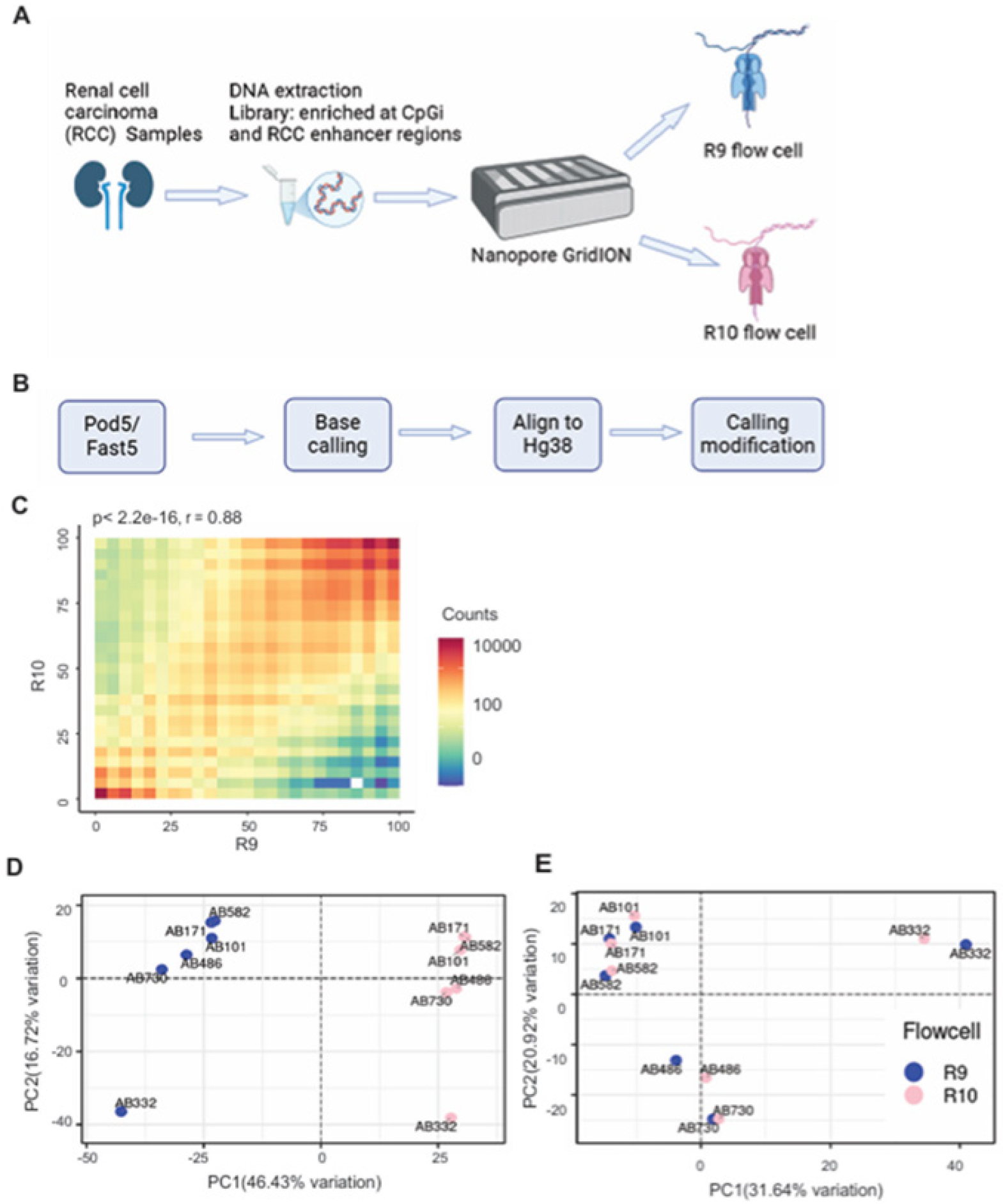
Systematic differences of R9 and R10 flow cells in detecting 5mC **A-B.** Schematic of the Nanopore long-read sequencing of genomic DNA from RCC patients using R9 and R10 flow cells (**A**), followed by bioinformatic data analysis (**B**). **C.** Pearson correlation analysis shows the correlation between 5mC detected by the R9 flow cell and that from the R10 flow cell. All the CpGs with coverage greater than 10 were included for analysis. **D-E.** PCA plots showing DNA methylation (5mC) in 6 pairs (R9 and R10) RCC samples before (**D**) and after batch correction (**E**).

To compare the performance of R9 and R10 flow cells in measuring DNA methylation (5mC), we isolated genomic DNA from 6 human renal cell carcinoma (RCC), with an equal number of African American (AA) vs. white patients and male vs. female patients (Table 1). We conducted reduced representation methylation sequencing (RRMS), targeting 523 Mb enriching for CpG islands, promoters, and prostatic enhancers, of all 6 samples using Nanopore GridION (Fig 1A). Each DNA sample was split for subsequent sequencing by both R.9 and R10 flow cells and the raw data were processed with the Nanopore standard pipelines (Fig 1B). We were able to obtain 5–11 million reads per sample reaching 8x to 24x coverage of target genomic regions. We detected over 23 million CpG sites in each sample, with methylated CpG sites varying between 70% and 81% among patients. Among the 12 samples, over 13 million CpG sites were shared, including 50,770 CpG sites with a minimum of 10× coverage, of which 84% with 5mC modifications. First, we performed Pearson correlation analysis of 5mC calls from R9 and R10 flow cells of the same sample and observed an overall good correlation (r=0.88, p<2.2e-16) (Fig 1C). However, Principal Component Analysis (PCA) of 5mC detected in all samples revealed two distinct clusters, separating R9 samples from R10 samples (Fig 1D), indicating drastic discordance of R9 and R10 flow cells in 5mC detection masking any biological differences among individual patients. We hypothesized that there were systematic differences in DNA methylation detection by R9 and R10 flow cells. To remove such effects, we utilized the Limma package[9] to remove batch effects on the 5mC data. Critically, PCA analyses the batch-corrected 5mC profiles clustered R9 and R10 samples of the same patients together (Fig 1E). In conclusion, our analysis revealed systematic bias between R9 and R10 flow cells in detecting DNA methylation that can be corrected using bioinformatics tools.

### R10 flow cells more accurately detect DNA methylation than R9 flow cells

**Figure 2.**
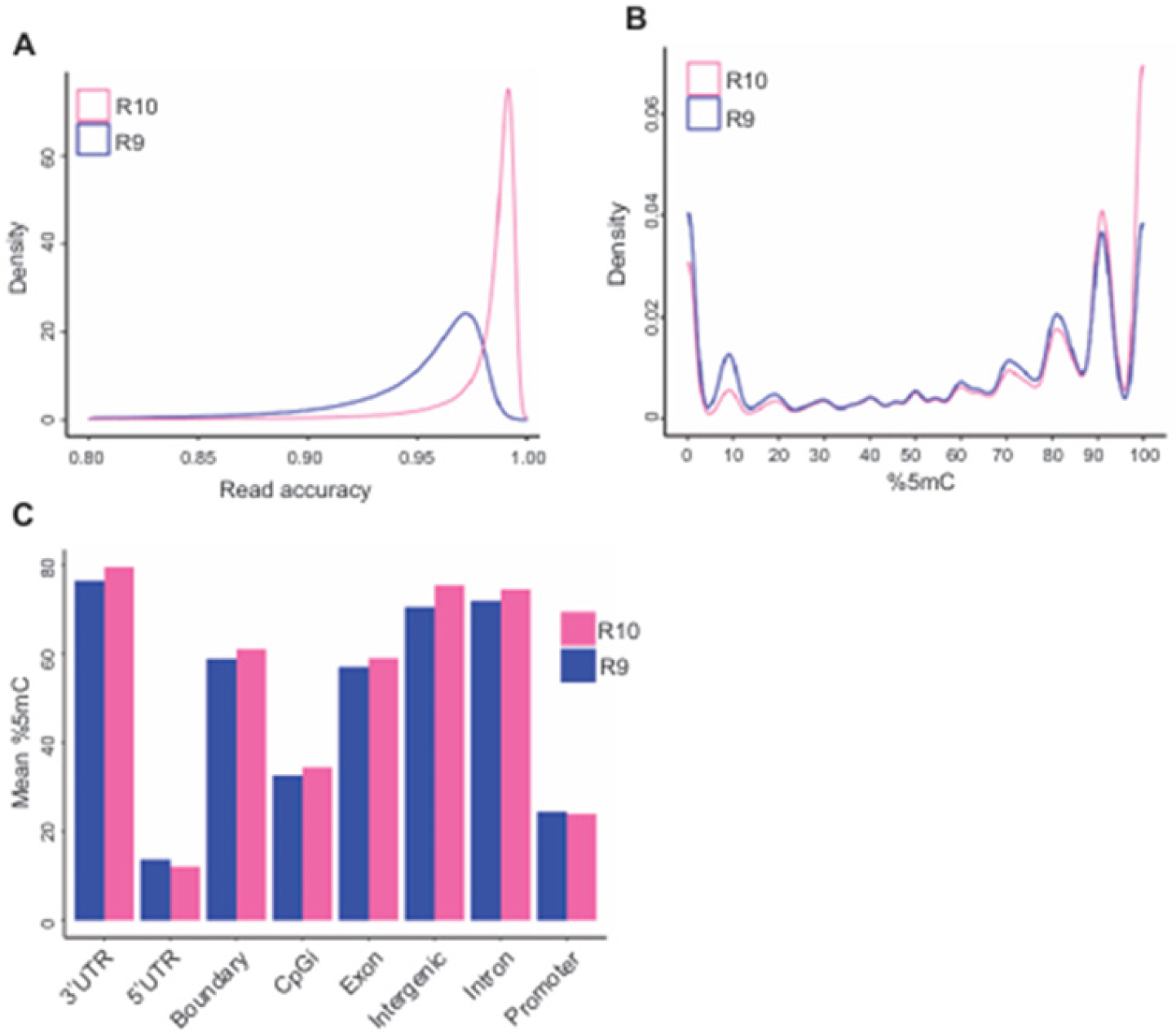
R10 flow cells more accurately detect DNA methylation than R9 flow cells. **A.** The density distribution plot shows the accuracy of reads compared to the reference genome. **B.** Density plot of DNA methylation level across all CpG sites detected by R9 and R10 flow cells in AB101. **C.** Mean DNA methylation profile of R9 and R10 flow cells at different genomic loci in sample AB101.

To further determine the potential mechanisms underlying the systematic biases in 5mC detection by R9 and R10 flow cells, we first examined their sequence accuracy by comparing sequence reads with reference reads, which may affect the accuracy in 5mC calling. Consistent with the previous study [8], the average sequence accuracy of R10.4 flow cells (97.1%) was much higher than that of R9.4.1 flow cells (93.1%) (Fig 2A). As repetitive DNA regions in the genome, which are the most challenging to sequence, are often heavily methylated [10], sequencing inaccuracy by R9 flow cells may impair its ability to accurately capture 5mC. We thus compared 5mC detection by R9 and R10 across CpG sites with different methylation levels. Compared with R10 flow cells, R9 flow cells detected significantly much less 5mC at CpG sites that were 100% methylated (Fig 2B). By contrast, R9 flow cells over-estimate 5mC at unmethylated CpGs, likely due to noisy background level of methylation. Concordantly, R9 flow cells detected less 5mC than R10 flow cells at 3’UTR, intron and intergenic regions that are often highly methylated, but under-estimated 5mC at 5’UTR, which often contains hypomethylated CpG islands (Fig 2C). In summary, our data demonstrates that, due to much higher sequence accuracy, R10 flow cell predicts 5mC much more accurately across the genome, including both hyper- and hypo-methylated regions.

### Differentially methylated regions (DMRs) due to flow cell types

**Figure 3.**
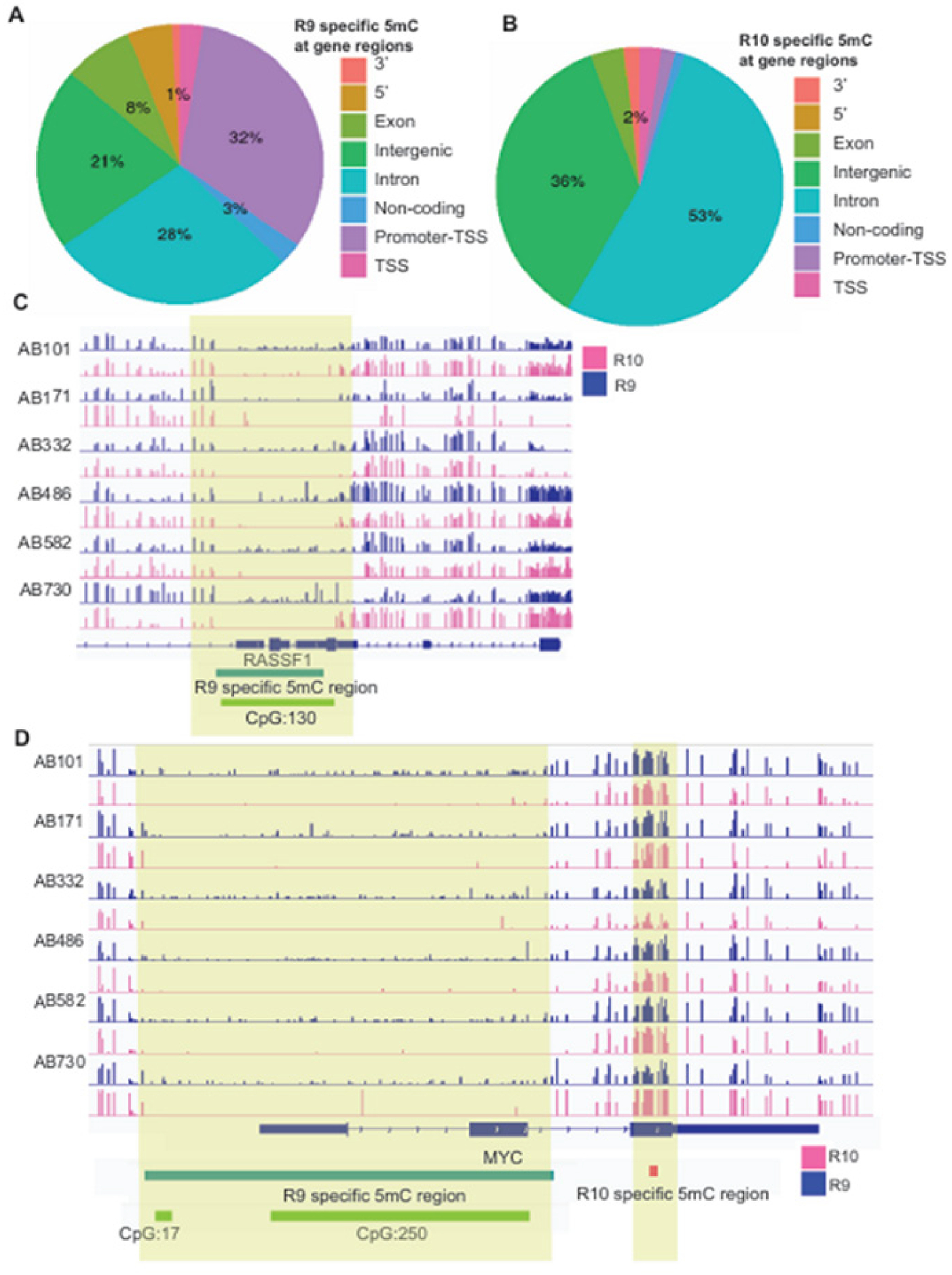
R9.4.1 and R10.4 flow cell-specific DNA methylations **A-B.** Genomic distributions of flow cell-specific DMRs: (**A**) R9-specific and (**B**) R10-specific. **C-D.** IGV showing DNA methylation profiles detected by R9 and R10 flow cells at RASSF1 gene locus (**C**) and MYC gene locus (**D**). The light-yellow shades indicate flow cell-specific DMRs. Green boxes under tracks indicate CpG islands.

To determine whether the lack of accuracy in 5mC calling by R9 can affect the detection of methylated regions, we performed differentially methylated regions (DMRs) analysis using the DSS package [11]. We defined DMRs with higher methylation in R9 and R10 as R9- and R10-specific DMRs, respectively. Only 7.9% of DMRs are R9-specific and the vast majority of DMRs are R10-specific. Interestingly, there were substantially more R9-specific DMRs (32%) than R10-specific DMRs (2%) at promoters, which often contain hypomethylated CpG islands (Fig 3A&3B). By contrast, a much higher percentage of R10-specific DMRs were found in introns (53%) and intergenic regions (36%) than in R9-specific DMRs (28% and 21%) (Fig 3A&3B). These findings are in agreement with our earlier observation that R9 over-estimated 5mC at promoters and under-estimated 5mC at intergenic regions (Fig 2C). Next, we specifically focused on DMRs at promoters, which are known to directly regulate gene transcription [12]. We found 13,795 DMRs at promoter regions, among these DMRs, 62.1% were R9-specific, and 37.9% regions were R10-specific, again supporting the notion that R9 over-estimated 5mC at promoters. For instance, closer examination of R9-specific DMR at the promoters of RASSF1 and MYC genes, which are known as oncogenes in RCC cancer[13, 14], showed that they were generally devoid of DNA methylation (Fig 3C&3D). However, R9 flow cells detected some basal methylation, which was likely caused by sequencing noise. In aggregates, comparing data from R9 and R10 flow cells might lead to artificial DMRs that were caused by sequence inaccuracy and noise in 5mC detection by R9.

## Discussion

The R10 flow cell has recently gained widespread adoption as a reliable and accurate platform for nanopore long-read sequencing. However, many researchers face the challenge of integrating datasets that include samples sequenced using both R9 and R10 flow cells. This raises major concerns about whether the research conclusion was driven by technical differences between flow cell types or real biological concepts.

In this study, we found a significant inconsistency of R9 and R10 flow cells in measuring 5mC in the same RCC sample. R9 flow cell detected a much higher 5mC level at regions of zero or no methylation, such as promoter CpG islands, which is likely due to a high noise-to-signal ratio caused by a lower sequencing accuracy. On the other hand, R9 flow cells under-estimated 5mC levels in chromatin regions of >90% methylated CpGs in the genome, such as intron and intergenic regions, which may be caused by the poor performance of R9 flow cells at homopolymeric DNA regions. R9 and R10 flow cells have different ‘readers’; the nanopore of the R9 flow cell only has a single reader in the middle of the barrel, whereas the R10 flow cell has two readers in the middle and increases the space and accuracy for sequencing.

We showed that such systematic differences could be largely removed through batch correction using the limma function. However, further studies are needed to determine whether this approach can correct all methylation differences arising from flow cell variations. We also want to note that our analysis did not extend to other epigenetic features, such as 5hmC modification, or structural variations, as they are beyond this study’s scope. As the intrinsic design of R10 and R9 flow cells are different, the inconsistency of both flow cells in detecting other epigenetic and genomic features may also be observed. For best data consistency, we suggest researchers use the same type of flow cells in one project.

## Materials and Methods

### Patient information

RCC tissue samples were obtained for research from patients consented under Emory protocol IRB00055316. Tumor tissue was frozen at −80°C freezer until use. The tumor histology for all patients was clear cell renal cell carcinoma at stage T3 or T4. Patient samples include: 1) 3 male and 3 female; and 2) three patients of African ancestry and three patients of European ancestry.

### Reduced Representation Methylation Sequencing (RRMS) library preparation

The RRMS libraries were performed using the protocol from Oxford Nanopore. Briefly, the genomic DNA of RCC samples was extracted with Quick-DNA Miniprep Plus Kit (Zymo, D4068) and fragmented to average size at 8 kb with g-TUBE^™^ (Covaris, 520079). The DNA libraries were prepared using a Ligation Sequencing Kit (SQK-LSK110) per the manufacturer’s protocol. The sequencing was performed on an Oxford Nanopore GridiON MK1 sequencer with R9.4.1 flow cells (FLO-MIN106D) or R10.4.1(FLO-MIN114) flow cells from Oxford Nanopore. The adaptive sampling (AS) method [15] was used for the targeted sequencing of regions of interest, enriching for CpG islands, shores, shelves, promoter regions, and RCC enhancers.

### Base and methylation calling

All ONT raw files were converted to pod5 files using pod5 v0.2.4 “pod5 convert fast5”.

The Dorado v0.9.0 basecaller command was used to basecall and align reads with hg38. The basecall with “SUP” models, with config file of “dna_r9.4.1_e8_sup@v3.3_5mCG_5hmCG@v0” for R9.4.1 and config file of “dna_r10.4.1_e8.2_400bps_sup@v4.3.0_5mCG_5hmCG@v1” for R10.4.1. We specified “modified based” as “5mC5hmC” to get modification information.

Example:

dorado basecaller

--reference “reference fasta” “config file”

--modified-bases 5mCG_5hmCG “pod5 file directory” > “output directory”

Bam files generated by Dorado were sorted and indexed using samtools v1.17.

CpG methylation was calculated using Modkit v0.2.4 from sorted bam files. The Modkit pileup command was used to create a bedMethyl format file for showing methylation status at the reference genomic(hg38) position. We filtered out 5mC with modification probability lower than the 10^th^ percentile, and only focused methylation at CpG sites.

Example:

modkit pileup --cpg –ref “reference fasta” “input sorted bam file” “output bed file”

### Differential methylation regions (DMR) analysis

DMRs were identified using the DSS (v 2.46.0) R package as previously reported [12]. Briefly, Bed-Methyl format files generated by Modkit were used as input. The smoothing span of 200bp was used in the DML test, and the data sequenced by R9.4.1 flow cells was used as a control. The DMRs were identified as below: The minimum length of DMRs was set at 100bp, with at least 5 CpG sites and more than 50% of CpG sites in this region being significantly methylated. For DMRs with lengths shorter than 50bp were merged with adjacent DMRs. Significant DMRs were defined with methylation change greater than 10%, and p-value less than 0.01. Principal component analysis (PCA) was generated by biplot from the R package PCA tools. Based on previous research, 10x coverage is reliable [35856633], and the minimum coverage of sequencing depth for PCA analysis, correlation test, density plot, and histography is 10.

## Figures and Tables

**Table1.** Clinical and sequence information of RCC samples.

Subject ID	Gender	Race	Recurrence	R9 Bases aligned	R9 CpG sites (% methylated)	R9 Coverage	R10 Base aligned	R10 CpG sites (% methylated)	R10 Coverage
AB101	Male	Black/AA	No	9,773,014,761	27,822,477 (80.3%)	20x	10,394,446,132	28272988 (80.2%)	19x
AB171	Female	White	Yes	11,075,270,756	39,860,319 (79.6%)	24x	4,640,766,034	23752508 (77.3%)	8x
AB332	Male	Black/AA	Yes	7,120,467,415	25,264,197 (70.1%)	15x	9,962,040,598	27407766 (73.5%)	18x
AB486	Female	Black/AA	Yes	10,186,328,053	27,141,978 (81.1%)	21x	6,376,290,751	25112461 (78.7%)	12x
AB582	Male	White	Yes	8,068,832,231	25,881,081 (80.0%)	17x	9,004,485,941	27182487 (80.8%)	17x
AB730	Female	White	Yes	7,851,290,817	26,317,188 (80.7%)	16x	4,717,117,029	23335203 (78.9%)	8x
